# Dataset for the life-cycle assessment of the Basque Y high-speed rail line in Spain

**DOI:** 10.1016/j.dib.2024.110472

**Published:** 2024-04-25

**Authors:** Andoni Kortazar, Gorka Bueno, David Hoyos

**Affiliations:** a Department of Public Policy and Economic History, Faculty of Business and Economics, Bilbao, University of the Basque Country UPV/EHU. Member of EKOPOL, Research Group on Ecological Economics & Political Ecology, Basque; b Department of Electronics Engineering, Faculty of Engineering, University of the Basque Country UPV/EHU. Member of EKOPOL, Research Group on Ecological Economics & Political Ecology, Basque; c Department of Quantitative Methods, Faculty of Business and Economics, Bilbao, University of the Basque Country UPV/EHU. Member of EKOPOL, Research Group on Ecological Economics & Political Ecology, Basque

**Keywords:** High-speed rail, Sustainable mobility, Life cycle assessment (lca), Environmental impact, Freight transport

## Abstract

This dataset presents a detailed description of the data and information used in the life-cycle assessment (LCA) of the Basque Y HSR line, which is a high-performance line for mixed traffic still under construction in 2023 (190 km). The LCI data presented in this paper support the original research carried out on whether the construction of the Basque Y HSR line infrastructure is justified in terms of reducing environmental impacts and energy consumption [1].

Life-cycle inventory (LCI) data related to the construction and maintenance phases of the infrastructure was collected using Google Earth tool following the information from stakeholder AHT gelditu [2], including the length of each item (bridges, tunnels, earthworks, railway tracks); and complemented with data obtained from the LCA carried out by Tuchschmid et al. [3]. LCI data associated with the operation phase of the infrastructure was built on passenger data for the years 2020, 2030, 2040 and 2049 available in ADIF [4], and freight data for the period of 2023–2050 available in the report by ADIF and the Basque Government [5]. Environmental impacts for transport modes were obtained from the ecoinvent v3.7 database [6,7] and processed with openLCA software [8]. Life-cycle impact assessment (LCIA) results gathered in the dataset include Global Warming (GWP100a), Cumulative Energy Demand and total emissions for PM10, SO_2_, NO_X_ and NMVOC.

Access to the explanation of these data allows any reader to reproduce the calculations of the main project and may be used as a baseline for future studies on transport economics too.

Specifications Table

**Table:** 

Subject	Transportation and environment
Specific subject area	Life-Cycle Assessment and rail transport
Data format	Raw and analysed data
Type of data	Text, tables, figures, Excel spreadsheet
Data collection	Raw data was acquired from primary and secondary data sources, including: (i) Google Earth, providing visual information related to the elements in the HSR line, and AHT gelditu [2]; (ii) public reports on passenger and freight traffic on the line in the future operation; (iii) LCA studies on other HSR infrastructures; and (iv) ecoinvent v3.7 commercial database. The line considered in the LCI was the Basque Y HSR still under construction in 2023. Primary data for the calculation of passenger and freight transport were obtained in pkm and tkm from public reports. Dynamic scenarios were analysed considering different combinations of occupancy rates and propulsion technologies for private cars, electricity mixes and future HSR transport demand in each decade. The description of the infrastructure elements of the HSR line was performed using Google Earth and its aerial perspective [2]. Complementary data for the LCI of the construction and maintenance phases was obtained from Tuchschmid et al. [3]. Passenger transport has been obtained from the information available in ADIF [4] and freight transport from a report by ADIF and the Government of the Basque Country [5]. Environmental impacts and material flows for modes of transport were collated from the ecoinvent v3.7 database [6,7] processed with openLCA v1.10 [8]. LCIA results were obtained for Global Warming (GWP100a) and Cumulative Energy Demand, along with total emissions for PM10, SO_2_, NO_X_ and NMVOC.
Data source location	Layout of the HSR line and all stations with high-speed services in mixed traffic (passenger and freight). Institution: ADIF Region: Euskadi (Basque Country) Country: Spain GPS coordinates of main stations: Bilbao-Abando: 43.2600321678635, −2.9285249607628336. Donostia-San Sebastián: 43.317710716449916, −1.9767595746112692. Vitoria-Gasteiz: 42.84158313012939, −2.672637849655704. Ezkio-Itxaso: 43.070977928726684, −2.2835783849376337. Irun: 43.33951902846531, −1.8012855368191591. Jundiz: 42.85157727968859, −2.7403785289776654. Astigarraga: 43.278653, −1.956859 Primary data sources (secondary data for this work): LCI of the construction and maintenance phases from AHT gelditu [2] and Tuchschmid et al. [3]. Passenger traffic data from ADIF and freight data from ADIF and the Government of the Basque Country report [4,5]. LCI data for modes of transport from the ecoinvent v3.7 database [6,7].
Data accessibility	Data are available within the article and detailed supplementary information is also provided through a repository. Repository name: Mendeley Data Data identification number: DOI: 10.17632/zm3hyp2shv.3 URL: https://data.mendeley.com/datasets/zm3hyp2shv/3
Related research article	Andoni Kortazar, Gorka Bueno, David Hoyos “A life-cycle assessment of the Basque Y high-speed rail project in Spain” (2023). Environmental Impacts Assessment Review URL: https://doi.org/10.1016/j.eiar.2023.107276

## 1 Value of the Data

• This dataset gathers a precise geographical description of the Basque Y HSR line infrastructure, including the measurements for each item, which can be crucial to other studies on this HSR project.
• The dataset provides necessary detailed life-cycle inventories to perform the life-cycle assessment of the Basque Y HSR line, but can be used as a baseline for researchers to perform an LCA of other HSR projects.
• This dataset may be used in future academic studies on transport economics, e.g. to calculate environmental footprints or economic profiles, and for any stakeholder interested in the environmental sustainability of HSR lines.
• This dataset provides a valuable reference for the evaluation of the environmental impacts of high-performance railway lines designed for mixed traffic (passengers at high speed and freight).

## 2 Background

Accessing comprehensive and reliable official data regarding high-speed rail transport in Spain presents a considerable challenge. Often, the data provided are insufficient for rigorous scientific inquiry, and even when requested, they are frequently withheld. Recognizing this opacity surrounding information, our aim is to enhance accessibility by providing readers with all the data and information utilized in the development of our article, ``Is high-speed rail a sustainable mobility option? A life-cycle assessment of the Basque Y project in Spain'', which was featured in the journal Environmental Impacts Assessment Review in 2023. Through this Data in Brief, we not only offer open access to the data but also provide detailed explanations, empowering readers to grasp the intricacies of our findings. Furthermore, these resources stand poised to support future investigations into high-speed rail, offering a valuable foundation for subsequent research endeavours.

## 3 Data Description

This dataset provides all primary and secondary data used to build the LCA of the Basque Y HSR line which is still under construction in 2023. The life-cycle inventory (LCI) data presented support the original research carried out on whether the construction of that infrastructure is justified in terms of reducing global warming effects and other environmental impacts and energy consumption, under two different dynamic scenarios (Optimistic scenario and Realistic scenario) [1].

Table 1 provides background data related to the scenarios considered. This background data includes not only the characterisation of the electricity mix that powers the high-speed rail infrastructure, but also the transport characteristics of the modes from which the infrastructure shifts passengers and freight, in a situation where the HSR infrastructure is not in operation.

**Table 1:** Background data related to the two scenarios considered (Optimistic and Realistic scenarios).

	2030–2039	2040–2049	2050–2059	2060–2069	2070–2079	2080–2090
**Optimistic Scenario (Passenger demand Freight demand**	5.84 million passengers 1.05 million tonnes	6.88 million passengers 2.38 million tonnes	7.60 million passengers 3.02 million tonnes			
**Realistic Scenario (Passenger demand** **Freight demand**	3.27 million passengers 0.59 million tonnes	3.85 million passengers 1.34 million tonnes	4.26 million passengers 1.70 million tonnes			

**Electricity mix**	100 % renewable;					

electricity from fossil fuels and nuclear removed from the mix in ecoinvent v3.7 (market for electricity, high voltage (ES))					

**Shifted transport from other modes**	Shifted passenger transport: from car 46.7 %, from bus 9.9 %, from conventional train 27.3 %, from aeroplane 4.5 % and 11.4 % for induced demand. Shifted freight transport: container ship 5 %, lorry 80 %, conventional freight train 10 % and induced demand 5 %. Calculated based on the information on transport demand provided by ADIF.					

**Passenger car occupancy rate**	1.68 passengers per car;			3.36 passengers per car;		
	average occupancy rate in Spain in 2014			occupancy rate doubles in comparison with that in the beginning of the operation		

**Car Propulsion**	28 % of cars are diesel and 22 % are petrol powered; statistical data from vehicle characteristics as specified by ecoinvent v3.7 Diesel vehicle: transport, passenger car, medium size, diesel, EURO 5 (RER) Petrol vehicle: transport, passenger car, medium size, petrol, EURO 5 (RER). 50 % of cars are of electric propulsion with renewable electricity; electricity from fossil fuels and nuclear removed from the mix.	100 % of cars are electric propulsion with renewable electricity; electricity from fossil fuels and nuclear is removed from the mix in ecoinvent v3.7 (market for electricity, high voltage (ES))				

**HSR operation**	Operation as specified by ecoinvent v3.7					
	transport, passenger train, high-speed (DE); with 100 % renewable electricity;					

	electricity from fossil fuels and nuclear removed from the mix (market for electricity, high voltage (ES))					

**Characteristics of other transport modes (aeroplane, coach, conventional train)**	Vehicle characteristics as specified by ecoinvent v3.7 for the following transport modes: Aeroplane: transport, passengers, aircraft, very short haul (GLO) Coach: transport, passenger coach (CH)					
	Conventional train: transport, passenger train (DE) ; with electricity: market for electricity, high voltage (ES)					

The data is structured in three parts: the first part (1.1) describes the LCI of the infrastructure's construction and maintenance phase; the second part (1.2) gathers the LCI of the operation phase and the third one (1.3) presents the LCIA data.

### 3.1 LCI on the construction and maintenance phase

The Basque Y HSR line will be part of the Northern Corridor of the Spanish HSR network (AVE) which will connect Spain and France at the west end of the border. In total, it will be almost 190 km in service by the time construction work is finished.

A detailed description of this HSR infrastructure, including the length of each item (bridges, tunnels, earthworks, railway tracks, passageways, forks, stations), is gathered in the A1 file (“A_1_Detailed_layout_inventory_of_the_HSR_line.doc”) and in the “Layout” tab of the C6 Excel file (“C_6_Basque_Y_HSR_ecoinvent_3.7.xlsx”).

The LCI for the construction and maintenance phase of the HSR line based on the LCI data provided by Tuchschmid et al. [3] and can be consulted at Kortazar et al. [9]. Here you can find the LCI of bridges, earthworks, tunnels, sleepers and ballasts, rails, mast, catenary and overhead wiring, signals and communication systems and construction and maintenance of railway buildings.

### 3.2 LCI of the operation phase

The LCI of the operation phase requires an accurate estimation of passenger transport over the Basque Y HSR line. This, in turn, requires knowledge of passenger and freight movements between stations as well as the distance travelled. Since the LCA is oriented towards the evaluation of the implementation of the HSR infrastructure, it is also necessary to compute the passenger and freight transport displaced from other modes to the HSR line, in comparison with an alternative scenario in which the infrastructure is not operative.

In the B1 file (“B_1_HSR_transport_demand_background_data.xlsx”), the “HSR Passenger Traffic” tab presents the passenger transport estimation for the 2020–2050 period provided by ADIF [4]. The “HSR Freight Traffic” tab presents data of freight movements in Jundiz as available in ADIF-GV [5]. All this data is primary transport demand data. The percentages of shifted passenger and freight transport from other modes to the Basque Y HSR have been taken from Kortazar [10], where they were calculated based on the information on transport demand provided by ADIF [4,11]. This information is gathered in the “Shifted transport” tab from the same Excel file.

The traffic data available in the B1 file makes it possible to obtain an estimation of the annual density of transport in the HSR in the entire useful lifetime of the project. The B2 Excel file (“B_2_HSR_transport_estimation.xls”) provides, as secondary transport demand data, the estimation for the passenger and freight transport in the HSR. In particular, this file provides the transport density of the HSR line (total transport over the line divided by the length of the line).

The B3 file (“B_3_Impact_factors_for_transport_modes_ecoinvent_v3.7.xlsx”) and the B4 file (“B_4_LCI_data_for_transport_modes_ecoinvent_v3.7.xlsx”) provide the LCI data for the transport modes considered in the operation phase of the HSR, obtained from the ecoinvent v3.7 database [6,7] and processed with openLCA software v1.10 [8].

Based on data from B3 and B4 files, Table 2 presents the LCI of the operation phase in of the Basque Y HSR in the period 2030–2039 in the Optimistic Scenario and Table 3 presents the LCI of the operation phase in of the Basque Y HSR in the period 2030–2039 in the Realistic Scenario. The B5 (“B_5_LCI_for_the_operation_phase_in_each_period_Optimistic_Scenario.doc”) and B6 (“B_6_LCI_for_the_operation_phase_in_each_period_Realistic_Scenario.doc”) files present all detailed LCI data of the operation phase of the Basque Y HSR line in each period of time in the Optimistic and Realistic scenarios, respectively.

**Table 2:** LCI for the operation phase of the Basque Y HSR in the period 2030–2039, in the Optimistic Scenario. Passenger transport is given annually and measured in passenger-kilometres (pkm) and freight transport is given annually and measured in tonne-kilometres (tkm).

Optimistic Scenario 2030–2039	Passenger transport	Global Warming	CED	PM10	SO_2_	NO_X_	NMVOC
Transport mode	pkm	gCO_2_eq·pkm^−1^	MJ·pkm^−1^	g·pkm^−1^	g·pkm^−1^	g·pkm^−1^	g·pkm^−1^
Passenger aircraft, very short haul	49,787,635.82	159.370	2.406	0.040	0.218	0.720	0.095
Passenger coach	110,173,083.27	49.440	0.817	0.032	0.055	0.467	0.052
Passenger car mix, 56 % diesel, 44 % petrol, 1,68 p/v	257,880,082.68	187.385	2.861	0.109	0.315	0.463	0.166
Electric car, REN, 1,68 p/v	257,880,082.68	56.304	1.399	0.099	0.179	0.175	0.069
Passenger train, Electricity ES-REN	301,375,267.85	27.120	0.734	0.035	0.054	0.215	0.029
Passenger train, high-speed, Electricity ES-REN	1,103,938,710.09	6.420	0.428	0.016	0.019	0.020	0.004

	Freight transport	Global Warming	CED	PM10	SO_2_	NO_X_	NMVOC
	tkm	gCO_2_eq·tkm^−1^	MJ·tkm^−1^	g·tkm^−1^	g·tkm^−1^	g·tkm^−1^	g·tkm^−1^

Transport, freight, sea, container ship	9,963,738.36	9.310	0.128	0.011	0.122	0.191	0.013
Transport, freight, lorry 16–32 t, EURO6	159,419,813.69	161.400	2.650	0.113	0.211	0.232	0.128
Transport, freight train, Electricity ES-REN	19,927,476.71	10.120	0.329	0.016	0.027	0.059	0.009
Freight train, high-speed, Electricity ES-REN	199,274,767.12	10.120	0.329	0.016	0.027	0.059	0.009

**Table 3:** LCI for the operation phase of the Basque Y HSR in the period 2030–2039, in the Realistic Scenario. Passenger transport is given annually and measured in passenger-kilometres (pkm) and freight transport is given annually and measured in tonnes-kilometres (tkm).

Realistic Scenario 2030–2039	Passenger transport	Global Warming	CED	PM10	SO_2_	NO_X_	NMVOC
Transport mode	pkm	gCO_2_eq·pkm^−1^	MJ·pkm^−1^	g·pkm^−1^	g·pkm^−1^	g·pkm^−1^	g·pkm^−1^
Passenger aircraft, very short haul	27,881,072.52	159.370	2.406	0.040	0.218	0.720	0.095
Passenger coach	61,696,918.79	49.440	0.817	0.032	0.055	0.467	0.052
Passenger car mix, 56 % diesel, 44 % petrol, 1,68 p/v	144,412,827.96	187.385	2.861	0.109	0.315	0.463	0.166
Electric car, REN, 1,68 p/v	144,412,827.96	56.304	1.399	0.099	0.179	0.175	0.069
Passenger train, Electricity ES-REN	168,770,128.56	27.120	0.734	0.035	0.054	0.215	0.029
Passenger train, high-speed, Electricity ES-REN	618,205,599.13	6.420	0.428	0.016	0.019	0.020	0.004

	Freight transport	Global Warming	CED	PM10	SO_2_	NO_X_	NMVOC
	tkm	gCO_2_eq·tkm^−1^	MJ·tkm^−1^	g·tkm^−1^	g·tkm^−1^	g·tkm^−1^	g·tkm^−1^

Transport, freight, sea, container ship	5,549,422.00	9.310	0.128	0.011	0.122	0.191	0.013
Transport, freight, lorry 16–32 t, EURO6	88,790,751.95	161.400	2.650	0.113	0.211	0.232	0.128
Transport, freight train, Electricity ES-REN	11,098,843.99	10.120	0.329	0.016	0.027	0.059	0.009
Freight train, high-speed, Electricity ES-REN	110,988,439.94	10.120	0.329	0.016	0.027	0.059	0.009

### 3.3 LCIA and total emissions data

LCIA results gathered in the dataset include Global Warming (GWP100a) and Cumulative Energy Demand. Total emissions to air of the elementary flows PM10, SO_2_, NO_X_ and NMVOC have also been considered in the analysis. The LCIA methods considered and the elementary flows for which final net balances have been analysed are the same as those considered by Kortazar et al. [9]. These indicators are aligned with those proposed by the EU manuals related to cost-benefit analysis and the evaluation of external costs of transport [12,13] that only add air emissions of NH3.

Table 4, Table 5, Table 6, Table 7, Table 8, Table 9 present data related to the processing of LCIA results for selected elementary fluxes and impact categories in the life cycle of the Basque Y HSR. Table 4, Table 5 of this document present the annual net emissions of the operation phase in each decade of the HSR project in the Global Warming impact category and in the Optimistic and Realistic scenarios. All these results for the rest of the elementary fluxes assessed are detailed in C1 file (“C_1_LCIA_results_and_annual_emissions_in_the_Optimistic_Scenario.doc”) for Optimistic Scenario and in C2 file (“C_2_LCIA_results_and_annual_emissions_in_the_Realistic_Scenario.doc”) for Realistic Scenario.

**Table 4:** Data related to the processing of LCIA results and annual net emissions of the operation phase in the Global Warming impact category and in the Optimistic Scenario. .

GLOBAL WARMING t/year of CO_2_eq	2030–2039	2040–2049	2050–2059	2060–2069	2070–2079	2080–2089
Operation passengers HSR	7087.29	8347.65	9219.93	9219.93	9219.93	9219.93
Operation freight HSR	2016.66	4556.63	5781.94	5781.94	5781.94	5781.94
Operation Shifted Aircraft	7934.66	9345.71	10,322.28	10,322.28	10,322.28	10,322.28
Operation Shifted Bus	5446.96	6415.62	7086.01	7086.01	7086.01	7086.01
Operation Shifted Car	48,322.98	0.00	0.00	0.00	0.00	0.00
Operation Shifted Electric car	14,519.57	34,203.31	37,777.35	18,888.67	18,888.67	18,888.67
Operation Shifted Train	8173.30	9626.79	10,632.73	10,632.73	10,632.73	10,632.73
Operation shifted container ship	92.76	209.60	265.96	265.96	265.96	265.96
Operation Shifted lorry 16–32	25,730.36	58,137.58	73,771.22	73,771.22	73,771.22	73,771.22
Operation Shifted freight train SPA	201.67	455.66	578.19	578.19	578.19	578.19

**Year Net Balance**	**−101,318.30**	**−105,489.98**	**−125,431.86**	**−106,543.19**	**−106,543.19**	**−106,543.19**

**Table 5:** Data related to the processing of LCIA results and annual net emissions of the operation phase in the selected Global Warming impact category and in the Realistic Scenario.

GLOBAL WARMING t/year of CO_2_eq	2030–2039	2040–2049	2050–2059	2060–2069	2070–2079	2080–2089
Operation passengers HSR	3968.88	4674.69	5163.16	5163.16	5163.16	5163.16
Operation freight HSR	1123.20	2552.73	3241.97	3241.97	3241.97	3241.97
Operation Shifted Aircraft	4443.41	5233.60	5780.48	5780.48	5780.48	5780.48
Operation Shifted Bus	3050.30	3592.74	3968.16	3968.16	3968.16	3968.16
Operation Shifted Car	27,060.87	0.00	0.00	0.00	0.00	0.00
Operation Shifted Electric car	8130.96	19,153.85	21,155.31	10,577.66	10,577.66	10,577.66
Operation Shifted Train	4577.05	5391.00	5954.33	5954.33	5954.33	5954.33
Operation shifted container ship	51.67	117.42	149.12	149.12	149.12	149.12
Operation Shifted lorry 16–32	14,330.83	32,570.07	41,363.99	41,363.99	41,363.99	41,363.99
Operation Shifted freight train SPA	112.32	255.27	324.20	324.20	324.20	324.20

**Year Net Balance**	**−56,665.30**	**−59,086.54**	**−70,290.47**	**−59,712.81**	**−59,712.81**	**−59,712.81**

Table 6, Table 7 present the evolution of the compensation balance for all the indicators throughout the useful life of the project in the Optimistic and Realistic scenarios. These results are provided on a yearly basis, assuming that the lifetime of the infrastructure is 60 years. The exact number of years of HSR operation required to compensate for the environmental impacts associated with the construction and maintenance of the infrastructure are also provided (“Compensation year”).

**Table 6:** Evolution of the compensation balance throughout the useful life of the project and the exact year of the compensation for all the indicators in the Optimistic Scenario.

Compensation Balance	Global Warming	CED	PM10	SO_2_	NO_x_	NMVOC
	CO2eq t/year	GJ/year	t/year	t/year	t/year	t/year
2030	2,446,823.27	25,403,290.50	2540.97	3842.98	6474.25	1001.82
2040	1,429,468.58	10,981,525.65	1847.62	2130.50	3242.45	48.75
2050	354,626.87	−5,576,268.88	910.32	194.92	−39.69	−993.56
2060	−880,803.10	−24,412,754.76	−131.19	−1959.69	−3662.41	−2172.16
2070	−1,946,235.02	−39,024,321.43	−873.17	−3573.14	−6757.37	−3141.23
2080	−3,011,666.93	−53,635,888.11	−1615.16	−5186.59	−3662.41	−4110.30

Compensation year	2053	2047	2057	2051	2050	2041

**Table 7:** Evolution of the compensation balance throughout the useful life of the project and the exact year of the compensation for all the indicators in the Realistic Scenario.

Compensation Balance	Global Warming	CED	PM10	SO_2_	NO_x_	NMVOC
	CO2eq t/year	GJ/year	t/year	t/year	t/year	t/year
2030	2,491,476.27	26,030,006.54	2570.41	3917.48	6616.51	1043.46
2040	1,922,402.01	17,963,609.09	2182.54	2959.30	4807.46	510.25
2050	1,320,332.65	8,688,843.44	1657.55	1875.17	2969.27	−73.57
2060	628,005.61	−1,866,977.19	1074.00	667.99	939.98	−733.97
2070	30,877.49	−10,056,842.96	658.18	−236.15	−793.77	−1277.03
2080	−566,250.64	−18,246,708.74	242.36	−1140.29	939.98	−1820.09

**Compensation year**	**2071**	**2059**	**2086**	**2068**	**2066**	**2049**

Table 8, Table 9 present the annual percentage reduction of impacts in the Optimistic and Realistic scenarios in year 2050 with respect to the impacts in the alternative scenario without the project, the first year the infrastructure is put into operation.

**Table 8:** Annual reductions in the Optimistic Scenario in year 2050, with respect to the baseline scenario without the project, the first year the infrastructure is put into operation.

	Global Warming	CED	PM10	SO2	NOx	NMVOC
2030–2039	24.5 %	25.0 %	15.1 %	24.8 %	62.2 %	38.8 %
2040–2049	26.3 %	30.2 %	31.6 %	30.0 %	62.4 %	42.8 %
2050–2059	34.6 %	38.0 %	41.4 %	37.6 %	73.2 %	51.8 %
2060–2069	26.7 %	26.0 %	19.9 %	23.0 %	58.9 %	40.0 %
2070–2079	26.7 %	26.0 %	19.9 %	23.0 %	58.9 %	40.0 %
2080–2090	26.7 %	26.0 %	19.9 %	23.0 %	58.9 %	40.0 %

**Table 9:** Annual reductions in the Realistic Scenario in year 2050, with respect to the baseline scenario without the project, the first year the infrastructure is put into operation.

	Global Warming	CED	PM10	SO2	NOx	NMVOC
2030–2039	5.91 %	8.91%	−3.95%	6.74%	27.69%	17.59%
2040–2049	6.92%	11.87%	5.29%	9.67%	27.81%	19.89%
2050–2059	11.59%	16.26%	10.85%	13.90%	33.85%	24.95%
2060–2069	7.18%	9.53%	−1.22%	5.72%	25.87%	18.32%
2070–2079	7.18%	9.53%	−1.22%	5.72%	25.87%	18.32%
2080–2090	7.18%	9.53%	−1.22%	5.72%	25.87%	18.32%

All this information is presented in figures in the C3 file (“C_3_Compensation_balance_and_annual_reduction_in_the_Optimistic_Scenario.doc”) for the Optimistic Scenario and in the C4 file (“C_4_Compensation_balance_and_annual_reduction_in_the_Realistic_Scenario.doc”) for the Realistic Scenario. Figures C.3.1–6 show the evolution of the compensation balance and annual reduction in the Optimistic Scenario for the GWP100 and the CED impact categories, and the PM10, SO2, NOx and NMVOC indicators. Figures C.4.1–6 show the evolution of the compensation balance and annual reduction in the Optimistic Scenario for the GWP100 and the CED impact categories, and the PM10, SO2, NOx and NMVOC indicators.

In the C5 file (“C_5_Impact_per_person_and_kilometre_of_different_modes_of_transport_in_each_scenario_and_period.doc”), the detailed environmental loads per passenger-kilometre for the different modes of passenger transport and for the HSR in the different decades are shown, broken down according to operating loads, and loads associated with the construction and maintenance of the infrastructure. Figures C.5.1–6 show the impact per passenger-kilometre of different modes of transport and of the transport by HSR in each scenario and period for the GWP100, CED, PM10, SO_2_, NOx and NMVOC impact indicators.

Given that the infrastructure is for mixed traffic (passengers and freight), the impact allocation has been carried out setting the energy consumption of each of type of transport (passengers, freight) as the allocation criterion for the impact distribution between passengers and freight transport. This information is also available in “Construction loads per pkm & tkm” tab of the C6 Excel file (“C_6_HSR_Y_BASQUE_ecoinvent_3.7.xlsx”).

## 4 Experimental Design, Materials and Methods

The purpose of compiling this dataset was to carry out an LCA of the Basque HSR line, in order to verify whether its construction is justified in terms of reducing environmental impacts and energy consumption and to verify whether this project will really help to meet the international objectives of mitigation of climate change. The secondary goal of the LCA was set to obtain and present the inventory data linked to the life-cycle of the infrastructure, also considering the environmental contribution of shifted transport of passengers and freight from other modes to the HSR. The scope of the study was the Basque HSR transport system, still under construction at the end of 2023. The functional unit was the passenger and freight transport service provided by the entire HSR line in one year of operation, which includes the passenger and freight services in the Basque Country. Primary data presented in the previous section was processed in a modelling that is described in the remainder of this section.

The net environmental impact (NetEI) linked to the functional unit, in comparison with a transport system in which the HSR is not operative, is calculated following Eq. (1):

(1) $$NetEI=\sum EI_{Construction\&Maintenance}^{HSR}+\sum EI_{Operation}^{HSR}-\sum_{i}EI_{i\to HSR}^{i}$$

where $EI_{Construction\&Maintenance}^{HSR}$ denotes the construction and maintenance impacts of the infrastructure, considering that they are evenly distributed along its lifetime (60 years) [14]; $EI_{Operation}^{HSR}$ denotes the impacts related to the annual operation of the HSR infrastructure; and $EI_{i\to HSR}^{i}$ denotes the impacts linked to the annual operation of alternative transport modes –referenced with the *i* subscript, i.e. aeroplane, conventional train, coach and private car for passenger transport; boat, conventional train and truck for freight transport – from which transport is shifted to the HSR.

Detailed information was needed on every kilometre of the Basque Y HSR infrastructure for the compilation of the inventory of the construction and maintenance phase. Faced with the fact that this project is still under construction and the impossibility of obtaining the construction plans for the line, the data was gathered using the Google Earth tool and based on the work developed by stakeholder “AHT gelditu!” [2]. A complete aerial review of every metre of the line was carried out, and all the measurements of the different items were recorded. The environmental impacts associated with the construction and maintenance of the HSR line were calculated assigning each item of the infrastructure the corresponding impact coefficient following Tuchschmid et al. [3]. This calculation is gathered in the “CONSTRUCTION IMPACTS MODELLING” tab of the corresponding C6 Excel file (“C_6_HSR_Y_BASQUE_ecoinvent_3.7.xlsx”).

The application of Eq. (1) to the primary and secondary data reported in Section 1 for the entire line is shown in the C6 Excel file. The environmental balance of the infrastructure has been calculated for each of the decades according to the background conditions indicated in Table 1 (the calculations for each decade are shown in the “OPTIMISTIC” and “REALISTIC” tabs of the Excel sheet), and then these scenarios have been combined into the full dynamic scenarios, the results of which are displayed in the “DYNAMIC OPTIMISTIC” and “DYNAMIC REALISTIC” tabs. These results are also presented through the annual percentage reduction of impacts in the Optimistic and Realistic scenarios with respect to the impacts in the alternative scenario without the project, in the first year that the infrastructure is put into operation. This reduction is calculated as the annual net balance in each decade divided by the total impacts associated with all the transport shifted to the HSR infrastructure from other modes. The information for the scenario without HSR is presented in the tab “Baseline Scenarios” of the C6 Excel file.

## Limitations

The most important limitation encountered in the process of preparing this study was that it was sometimes difficult to obtain data on transport demand. Therefore, in order to solve this problem, it would help if the administrations provided transport information in the most transparent and rigorous way possible.

## Ethics Statement

The authors have read and follow the ethical requirements for publication in Data in Brief and confirming that the current work does not involve human subjects, animal experiments, or any data collected from social media platforms.

## CRediT authorship contribution statement

**Andoni Kortazar:** Writing – original draft, Writing – review & editing, Investigation, Software. **Gorka Bueno:** Conceptualization, Writing – review & editing, Methodology, Software. **David Hoyos:** Writing – review & editing, Supervision.

## Data Availability

Data of the LCA Basque Y HSR (Original data) (Mendeley Data).

## References

[1] Kortazar A., Bueno G., Hoyos D. (2023). «Is High Speed Rail a sustainable mobility option?. A Life Cyc. Assessm. Basque Y HSR Project Spain».

[2] gelditu A.H.T. (2019). «AHT Gelditu-Paremos el TAV: TAV en Euskal Herria». AHT Gelditu-Paremos el TAV.

[3] M. Tuchschmid, W. Knörr, A. Schacht, M. Mottschall, M. Schmied, Carbon Footprint and environmental impact of Railway Infrastructure. International Union of Railways, UIC, 20 de octubre de 2011 (2011). [En línea]. Disponible en: https://uic.org/IMG/pdf/uic_rail_infrastructure_111104.pdf.

[4] ADIF, «Estudio de rentabilidad económico-social y financiera de la Línea de Alta Velocidad Vitoria-Bilbao-San Sebastián e implantación del ancho estándar UIC en los tramos Burgos-Vitoria y Astigarraga-Irún», jun. 2015. Accedido: 1 de diciembre de 2018. [En línea]. Disponible en: https://goo.gl/p9ZhTw

[5] ADIF-Gobierno Vasco, «Estudio de demanda de mercancías para la terminal logística de Jundiz.» febrero de 2019.

[6] Wernet G., Bauer C., Steubing B., Reinhard J., Moreno Ruiz E., Weidema B. (sep. 2016). «The ecoinvent database version 3 (Part I): overview and methodology». Int. J. Life Cycle Assess.

[7] Ecoinvent, «ecoinvent 3.7», 2020. https://www.ecoinvent.org/database/ecoinvent-371/ecoinvent-37/ecoinvent-37.html (accedido 16 de febrero de 2021).

[8] Ciroth A. (jun. 2007). «ICT for environment in life cycle applications openLCA — a new open source software for life cycle assessment». Int. J. Life Cycle Assess.

[9] Kortazar A., Bueno G., Hoyos D. (jun. 2021). «Dataset for the life-cycle assessment of the high-speed rail network in Spain». Data Brief.

[10] Kortazar A. (2021). https://addi.ehu.es/handle/10810/55515.

[11] ADIF, «Estudio de rentabilidad económico-social y financiera de la Línea de Alta Velocidad Vitoria-Bilbao-San Sebastián y tramo en 3 carriles Astigarraga-Irún», feb. 2015.

[12] (2015). European Commission y Directorate-General for Regional and Urban Policy, *Guide to cost-benefit analysis of investment projects : economic appraisal tool for cohesion policy 2014-2020*. Public. Office.

[13] Commission European (2020). Handbook on the external costs of transport : version 2019 –1.1. Public. Office.

[14] R. Hischier et al., «Implementation of Life Cycle Impact Assessment Methods», p. 176, 2010.

